# Editorial: Psychometrics in Psychiatry 2022: Autism

**DOI:** 10.3389/fpsyt.2024.1416556

**Published:** 2024-06-05

**Authors:** Flavia Lecciso, Antonio Narzisi

**Affiliations:** ^1^ Department of Human and Social Science, University of Salento, Lecce, Italy; ^2^ Lab of Applied Psychology, Department of Human and Social Science, University of Salento, Lecce, Italy; ^3^ Department of Child Psychiatry and Psychopharmacology, IRCCS Stella Maris Foundation, Pisa, Italy

**Keywords:** autism, psychometrics, neurodevelopmental disorders, screening, daily living skills, concerning behavior, work skills

The currect Editorial aims at summarizing the topics discusse by the published papers in this Research Topic on measures for screening and assessment fo Autism Spectrum Disorders (ASD). “*Psychometrics in psychiatry 2022: autism*” is an interesting project that has adopted a scientific point of view with important implications for research and clinical practice. Five contributions are included in this Research Topic from different cultural backgrounds, i.e., Iran, the United States (two papers), Japan, Kenya, and the United Kingdom. Worldwide, the articles involved 38 authors and 11 expert reviewers working in the field of ASD and neurodevelopmental disorders.

Overall, the prevalence of ASD (1) encourages the design of studies to validate tools for assessing autism and its related difficulties. Systematic reviews (2–4) have highlighted the promising psychometric properties of existing screening measures that have been applied in different Western cultures (e.g., 5–9). Unfortunately, screening efforts are lacking on continents such as Africa. The contribution by Kipkemoi et al. on Kenyan children aged 6–9 years, aims to develop an interview to identify and diagnose autism.

The study by Wada et al. developed an easy and user-friendly tool to assess sensory issues in autism. This feature is also common to other neurodevelopmental disorders, such as attention-deficit/hyperactivity disorder (ADHD; 10) and specific learning disorder (SLD; 11). Qualitative and quantitative analyses of this novel tool with 6-70-year-old individuals showed successful preliminary outcomes, highlighting the importance of evaluating this autism-related difficulty during the diagnostic procedure.

In addition, this Research Topic includes studies that validate measures that not only reflect the two main core diagnostic criteria for autism (i.e., social/communicative difficulties; the presence of restricted interests, and repetitive behaviors), but also assess autism-related difficulties that negatively impact the individual with ASD and his or her family and, as a result, require targeted and meaningful interventions (12). The study by Mohammadi et al. validated a measure evaluating mental health and problem/risky behaviors in children and young individuals with ASD; the study by Uljarević et al. developed and validated a tool examining daily living skills. More specifically, the study by Mohammadi et al. examined the psychometric validity of the Assessment of Concerning Behavior (ACB) in the Iranian population revealing a good factor structure and supporting the comorbidity with internalizing (intrapersonal) and externalizing (interpersonal) problems, which are two key areas to monitor to promote the well-being of individuals (13). The study by Uljarević et al. aimed to develop and validate a novel open-source and low-cost measure that assesses daily living skills (in the form of personal, household, and community skills) in children and adolescents from 2 to 17 years old. The findings reported the he measure’s excellent potential for adoption in research and clinical settings.

Finally, the work submitted by Smith et al. analyzed the psychometric properties of an interview designed to assess work skills in young adults with ASD. The investigated measure has relevant research and clinical implications, in an increasingly current perspective in which employment is a key factor for a good quality of life (14), especially during the transition to young adulthood (16–26 years old; 15).

In conclusion, the Editors are grateful to all the Authors for submitting their work, and to the Reviewers for dedicating their time to improving the quality of the published manuscripts.

## Author contributions

FL: Conceptualization, Writing – original draft, Writing – review & editing. AN: Writing – review & editing.
